# *Toxoplasma gondii* in sheep: Serological occurrence at slaughterhouse level in Italy and environmental risk factors

**DOI:** 10.3389/fvets.2023.1057277

**Published:** 2023-03-23

**Authors:** Roberto Condoleo, Pasquale Rombolà, Roberta Palumbo, Davide Santori, Salvatore Serra, Sara Tonon, Antonio Bosco, Erminia Sezzi

**Affiliations:** ^1^Istituto Zooprofilattico Sperimentale del Lazio e Toscana, Rome, Italy; ^2^Viterbo Local Veterinary Services, Viterbo, Italy; ^3^Department of Veterinary Medicine, Federico II University, Naples, Italy

**Keywords:** *Toxoplasma gondii*, sheep, geographic information systems, environmental factors, bioclimatic variables

## Abstract

Toxoplasmosis is a parasitic disease affecting a wide range of species, including humans, and can be responsible for important clinical manifestations such as abortion and neurological signs. Sheep show a remarkable susceptibility to its causative agent, *Toxoplasma gondii*, and zoonotic transmission may occur in case of consumption of undercooked meat obtained from infected animals. *Toxoplasma gondii* seroprevalence in sheep can significantly vary on a geographical basis, as shown by numerous surveys conducted worldwide. To investigate environmental and climate conditions that may affect the likelihood of ovine infection, 405 serum samples from selected sheep raised in 91 farms were collected from two abattoirs, with each abattoir receiving animals from two regions (1/Tuscany-Latium and 2/Campania-Basilicata). The seroprevalence of infection in all examined animals was 53.8%. Young animals (*n* = 165) had a lower likelihood of being *T. gondii* positive compared to the adults (OR = 0.21), and the seropositive rate of animals slaughtered in abattoir 2 was significantly higher than that of animals slaughtered in abattoir 1 (60.5 vs. 43.2%, *p* < 0.01). The significant bioclimatic variables (*p* < 0.05) associated with the presence of *T. gondii* antibodies were related to areas with a lower range of temperature and higher precipitation. In conclusion, this study expands on the interpretation of serological data, with the inclusion of environmental and climatic variables, as possible risk factors in the spread of toxoplasmosis in the study area. These findings provide novel insights to support public health measures, such as risk-based control plan, and contribute to a “One Health” approach, taking into account the environmental and climatic perspectives.

## 1. Introduction

Toxoplasmosis is one of the most common parasitic zoonoses worldwide and its causative agent, *Toxoplasma gondii*, has a cosmopolitan distribution (1).

Human toxoplasmosis might occur through the ingestion of tissue cysts by consumption of undercooked meat of infected farmed animals, such as small ruminants, pigs, poultry, and horses, or by ingestion of oocysts sporulated in the environment, deriving from feline feces. Toxoplasmosis is therefore food-borne, water-borne, and soil-borne zoonosis (2). Among domestic species, sheep tend to be particularly susceptible to parasite infection. These animals are particularly exposed to the oocysts spread in the environment by felids, the definitive hosts of the parasite (3). As sheep are commonly raised through the extensive system, they graze outdoors frequently and, consequently, may be frequently exposed to the parasite. In case of infection, sheep can manifest clinical symptoms, especially if they are gravid, such as abortion, miscarriage, and fetal malformations. After the acute phase, *T. gondii* tends to localize in muscle and/or cerebral tissues where it forms cysts that contain the bradyzoites, the chronic form of the parasite. Bradyzoites might remain infectious for a long period and, when ingested by a host (humans included), repeat this asexual cycle (the sexual cycle is possible only in the definitive host). Ingestion of cysts by consumption of meat from lamb or mutton has been identified as a possible cause of infection in humans (4).

The occurrence of sheep toxoplasmosis can vary significantly on the basis of numerous factors, including the geographic location of the farm (3). In Italy, several surveys were performed in the past to assess the serological prevalence of *T. gondii* in the sheep population. Although differences due to the different analytical methods and study design are prevalent, the presence of *T. gondii* antibodies has been usually found in a relevant proportion of the tested sheep, implying that toxoplasmosis was not uncommon in this country (5–10). However, most of these studies have been conducted on circumscribed territories (one province or region); therefore, their results (i.e., seroprevalence estimates and evaluation of the risk factors detection) should mainly refer to the specific studied areas.

Environmental and climatic conditions may affect the survival of *T. gondii* in the environment and the subsequent risk of exposure for grazing animals. Environmental characteristics of the farm/grazing area, i.e., the presence and type of available water sources, the presence of certain animal species, or altitude, have been associated with a higher seropositivity rate in some countries (11–15). Climatic conditions of an area may be responsible for the length of the oocysts' infectious period since they survive within certain temperatures or moisture ranges (16). Nevertheless, to date, only one research has explored the association between *T. gondii* infection and environmental variables in Italy (17); however, no studies concerning the impact of climate have been conducted in this country as yet.

The present study aimed to (i) collect updated data regarding the serological occurrence of *T. gondii* in sheep from different regions of Italy and (ii) investigate the potential risk factors with a focus on variables related to environmental and climatic conditions.

## 2. Materials and methods

### 2.1. Study design

The study was conducted over a period of 21 months (between October 2019 and June 2021) and the sampling was carried out in two slaughterhouses located in two different regions of Central-Southern Italy (Latium and Campania). The sample size was calculated on the basis of an expected prevalence of 50%, α = 5%, 95% CI (*n* = 385 sheep). The overall number of animals to be tested was divided equally between the adults (age >12 months) and the young animals/lambs (age <12 months). Only the adult animals raised on the same farm for at least 6 months were selected, while the young animals/lambs had to be born and raised on the same farm of origin. The number of animals tested for each sampling session depended on the number of sheep that met the mentioned criteria and the available laboratory capacity (convenience sampling). The number of samples to be collected was equally distributed among the batches of sheep at the slaughterhouse on the day of sampling (a batch was meant as a group of animals delivered by the same farm). Animals to be tested were randomly chosen from within the batch. Information about the age of the animal, the name of the farm of origin, and its location was recorded using specific forms.

### 2.2. Serological analysis

The serum was extracted from all blood samples that were collected at the two abattoirs and tested using an enzyme-linked immunosorbent assay (ELISA) to detect the *T. gondii* antibodies. Tests were performed by the ELISA commercial kit, ID Screen^®^ Toxoplasmosis Indirect Multi-species (IDVET, Grabels, France) according to the manufacturer's instructions. To define the results, the following equation was used:


$$\begin{array}{l} Value\;\%=\;\frac{\left(OD\;sample-OD\;negative\;control\right)}{\left(OD\;positive\;control-OD\;negative\;control\right)}\;\times 100 \end{array}$$

If the test sample's percentage value was ≤50%, the test was considered to be positive. If the result was ≤40% and <50%, the result was uncertain. Finally, if the result was <40%, the test was considered to be negative.

### 2.3. Geographic information system

On the basis of literature and data availability, some environmental and demographic variables were chosen to be evaluated as the possible risk factors in the spread of toxoplasmosis in the study area (intended as the area where the farms of origin were located): slope, aspect, altitude, distance from water bodies, soil classification, land use/cover, domestic cat population density, and climate variables.

Data on the elevation, slope steepness, and aspect (slope direction) were obtained from a digital elevation model (DEM) having a 20-m spatial resolution (ISPRA, Sinanet, http://www.sinanet.isprambiente.it/it/sia-ispra/download-mais/dem20/view). This aspect was divided into the following eight classes: North (337.5–360° and 0–22.5°), North-East (22.5–67.5°), East (67.5–112.5°), South-East (112.5–157.5°), South (157.5–202.5°), South-West (202.5–247.5°), West (247.5–292.5°), and North-West (292.5–337.5°). The slope steepness was divided into the following four classes: flat (<1°), low (1–10°), medium (10–20°), and high (>20°).

Data on the land use/cover of the study area were obtained by the Corine Land Cover (CLC) map (2018 version Version 2020_20u1; European Environment Agency, Copenhagen, Denmark, https://land.copernicus.eu/pan-european/corine-land-cover/clc2018). CLC has a spatial resolution of 100 m. It is based on the classification of satellite images into a hierarchical structure of three levels and 44 classes. We used level 1 in this study. It includes five classes (artificial surfaces, agricultural areas, forests and seminatural areas, wetlands, and water bodies).

The distance from the closest water body (a detailed river network or other water bodies, European catchments and Rivers network system (Ecrins), https://www.eea.europa.eu/data-and-maps/data/european-catchments-and-rivers-network) was calculated for each farm with the “Near” tool of ArcGIS (ESRI 2020. ArcGIS Desktop 10.8.1).

To explore the possible influence of the clay-sand soils [as in (2)], a layer with general pedological categories (FAO soil classification, 1974) was used.

The density of the domestic cat population (n. cats /km^2^) refers to the number of cats that are present in proximity to the farm. This number was estimated in relation to the human population living in the surrounding area using the human:cat ratio (as reported in the study of Carvelli et al. 18). Human population values (ISTAT Census 2011) were extracted for each cell where the farm was located from the raster layer (1 km of spatial resolution) ISTAT-Eurostat (https://ec.europa.eu/eurostat/web/gisco/geodata/reference-data/population-distribution-demography).

Regarding climate data, 19 bioclimatic variables were obtained from the WorldClim spatial dataset (www.worldclim.org) at the highest available spatial resolution of 30 s (~1 km^2^) (Table 1). These were monthly mean data for the period 1970–2000.

**Table 1:** *Taxoplasma gondii* seropositive rate by bioclimatic variable.

**Variable**	**Negative, *N* = 182^1^**	**Positive, *N* = 218^1^**	***p*** **-value^2^**
BIO1 Annual mean temperature (°C)	13.5 (11.7; 15.4)	13.5 (11.4; 15.4)	0.79
BIO2 Mean diurnal range [mean of monthly(max temp - min temp)] (°C)	9.1 (7.6; 10.0)	8.5 (6.9; 10.2)	0.001^***^
BIO3 Isothermality (BIO2 / BIO7) (× 100)	33.6 (30.2; 37.7)	32.8 (29.6; 37.6)	0.014^*^
BIO4 Temperature seasonality (standard deviation × 100)	665.4 (600.7; 698.5)	661.4 (580.6; 697.9)	0.12
BIO5 Max temperature of warmest month (°C)	26.6 (23.9; 28.8)	26.6 (24.3; 29.0)	0.88
BIO6 Min temperature of coldest month (°C)	−0.3 (−1.8; 3.2)	−0.2 (−1.8; 3.9)	0.45
BIO7 Temperature annual range (BIO5-BIO6) (°C)	26.9 (24.3; 27.9)	26.3 (22.7; 28.1)	0.001^***^
BIO8 Mean temperature of wettest quarter (°C)	10.1 (8.5; 16.0)	10.1 (8.1; 13.7)	0.17
BIO9 Mean temperature of driest quarter (°C)	22.0 (20.2; 23.2)	21.9 (19.7; 23.2)	0.29
BIO10 Mean temperature of warmest quarter (°C)	22.0 (20.2; 23.2)	21.9 (19.7; 23.2)	0.32
BIO11 Mean temperature of coldest quarter (°C)	5.8 (4.0; 8.6)	5.8 (3.7; 8.9)	0.95
BIO12 Annual precipitation (mm)	602.0 (330.0; 805.0)	614.0 (330.0; 859.0)	0.12
BIO13 Precipitation of wettest month (mm)	81.0 (42.0; 113.0)	78.0 (42.0; 117.0)	0.19
BIO14 Precipitation of driest month (mm)	20.5 (8.0; 28.0)	21.0 (7.9; 28.0)	0.65
BIO15 Precipitation seasonality (coefficient of variation)	32.5 (26.5; 46.0)	35.7 (27.3; 46.8)	0.002^**^
BIO16 Precipitation of wettest quarter (mm)	218.0 (114.0; 308.0)	211.0 (114.0; 320.0)	0.14
BIO17 Precipitation of driest quarter (mm)	80.0 (45.0; 121.0)	80.0 (45.0; 99.0)	0.7
BIO18 Precipitation of warmest quarter (mm)	83.5 (45.0; 121.0)	83.0 (48.0; 119.0)	0.53
BIO19 Precipitation of coldest quarter (mm)	177.0 (88.0; 268.7)	184.0 (88.0; 269.1)	0.021^*^

1 Median (5%, 95%).

2 Kruskal–Wallis rank sum test.

* <0.05

** <0.01

*** 0.001.

### 2.4. Statistical analysis

Data collected during the sampling sessions, GIS information, and results of the serological tests were recorded in an Excel spreadsheet (version 2016, Microsoft Corporation, Redmond, WA, USA) and were used to carry out a descriptive statistical analysis using WinEpi and R (R version 4.1.3). Qualitative data were described using frequencies and percentages (Clopper–Pearson confidential intervals). After excluding the subjects with uncertain results, non-parametric statistical tests (Pearson's chi-squared test and Fisher's exact test) were preliminarily used to study the relationship between antibody seroreactivity toward the parasite and both animal-related (age category, size of the farm, and abattoir of provenience) and environment-related categorical variables (slope, aspect, altitude, distance from water bodies, soil classification, land use/cover, and domestic cat population density). Then, these mentioned variables were initially included in a generalized linear mixed-effects model fit by maximum likelihood using the package *lme4* (https://CRAN.R-project.org/package=lme4, version 1.1–31) from R statistical software (https://www.r-project.org/; version 4.4.1). The serological status of the single animals (i.e., “positive” or “negative”) was the dependent variable, while the variable “farm” was included as a random effect, considering that individual animals were clustered in farms. The function *dredge* from R package *MuMIn* (https://cran.r-project.org/web/packages/MuMIn; version 1.47.1) was used to select the combinations of variables that generated the top model set on the basis of the Akaike Information Criterion [ΔAIC value < 2)]. The model averaging approach was adopted to summarize the results and to estimate the relative importance of the predictors (R package *MuMIn*, functions *model.avg* and *sw*) (18). Explanatory variables were tested for multicollinearity, calculating the variance inflation factor (VIF) on the initial model (R package *car*, https://cran.r-project.org/web/packages/car/), and a value >10 was considered the cutoff point. The results were represented using odds ratios and 95% CIs. The association between bioclimatic variables (continuous data) and *T. gondii* seropositivity was explored using the Kruskal–Wallis rank sum test. Associations between variables were considered significant when *p*-values were lower than 0.05.

## 3. Results

**Figure 1:**
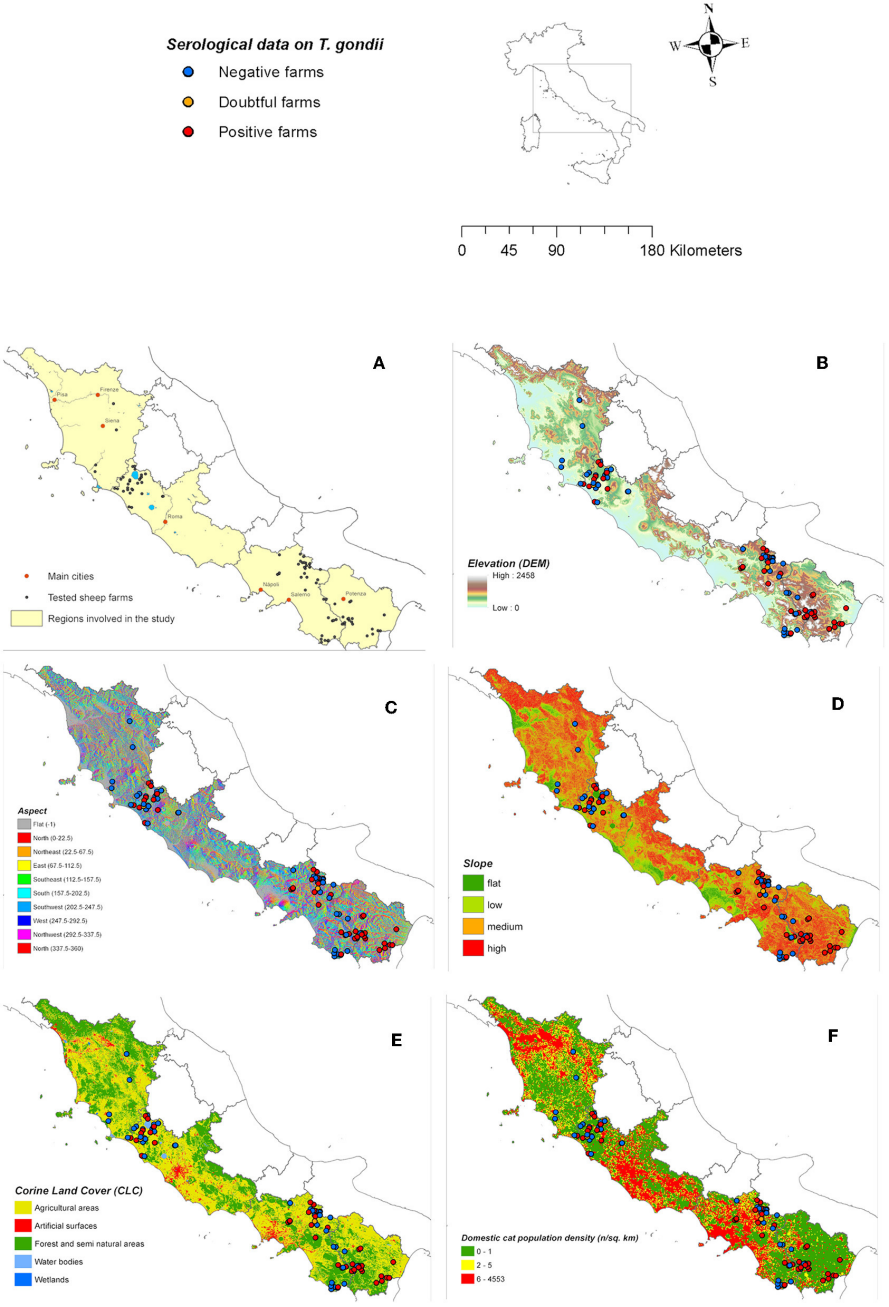
A map of serological results and location of the farms included in the investigation **(A)** presented by elevation **(B)**, predominant aspect **(C)**, slope steepness **(D)**, Corine Land Cover classes **(E)**, and estimated domestic cat population density **(F)** in the study area. The positive farm was defined on the basis of at least one positive serological result.

A total of 405 blood samples were collected at the two slaughterhouses, located in two Italian regions (Latium = 189, Campania = 216), during 33 different visits. The collected number of sheep samples per session varied between 1 and 34 on the basis of the availability of heads that met the sample criteria (see Material & Methods). The selected sheep were raised in 91 farms located in four regions, namely Basilicata (*n* = 17), Campania (*n* = 40), Latium (*n* = 30), and Tuscany (*n* = 4) (Figure 1). The size of the sheep farms ranged between 5 and 1,382 animals with a mean of 269 animals per farm.

**Figure 2:**
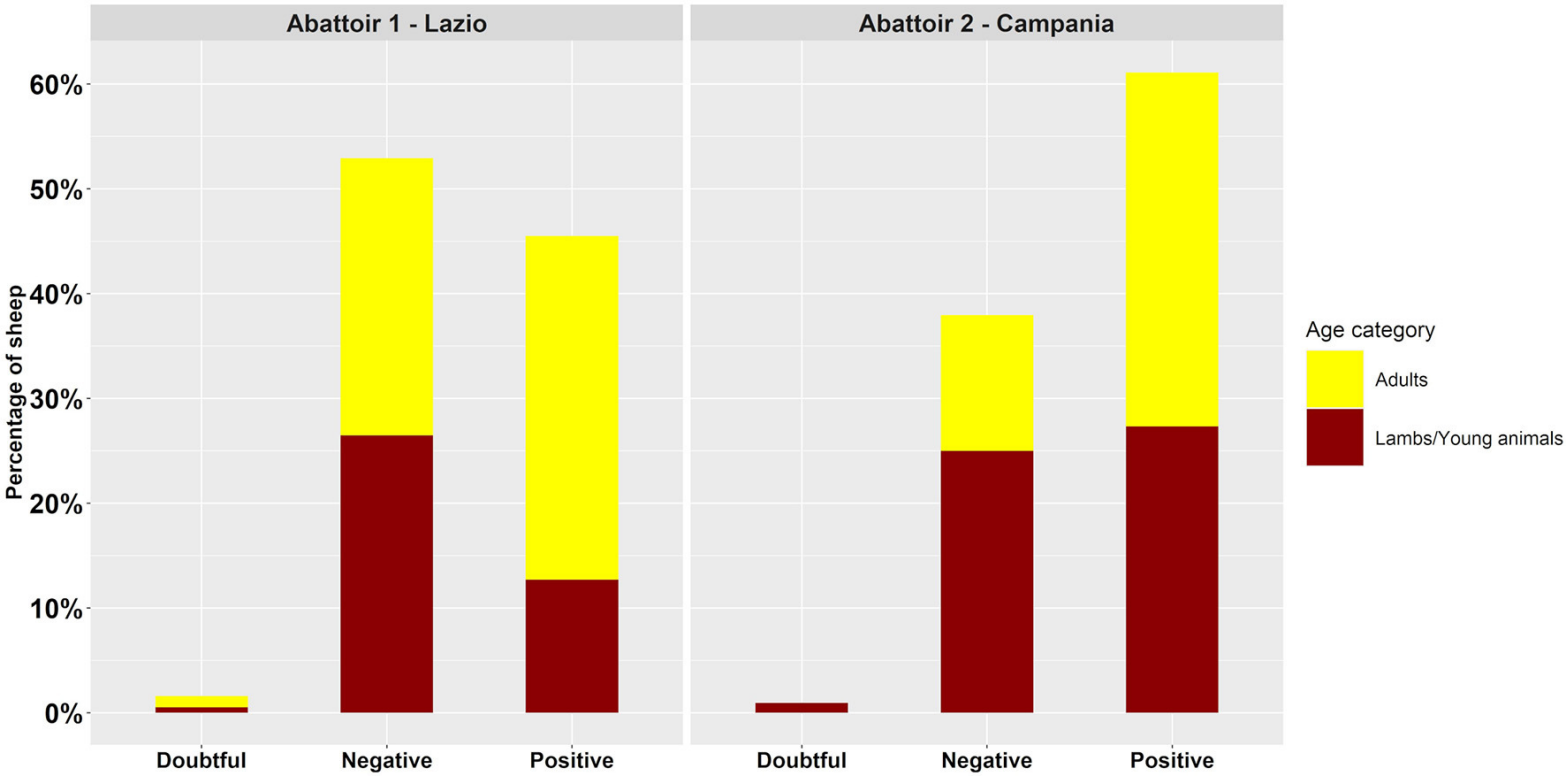
Serological test results by age category and abattoir.

Overall, approximately half of the animals became serologically reactive against *T. gondii* (218/405, 53.8%, CI 95% [48.9 to 58.9%]), although the positivity rate was significantly higher for adult animals (135/215, 62.8%, CI 95% [56 to 69.3%]) than for lambs/young animals (83/190, 43.7%, [36.5 to 51.1%] CI 95%) (*p* < 0.001). The seropositive rate of animals slaughtered in abattoir 2 (Campania region) was significantly higher than that of animals slaughtered in abattoir 1 (Latium region) (*p* < 0.01) (Figure 2).

After using the model selection approach, a top model set including 10 models was determined from 1,024 generated candidates. The risk factors with the highest relative importance were the age category and the abattoir, followed by the farm size. Model-averaged parameter estimates and the relative importance of predictors are reported in Table 2. Multicollinearity was not detected. The results of the univariate analysis were reported in the Supplementary material.

**Table 2:** Multinomial logistic regression analysis of risk factors associated with *Toxoplasma gondii* infections in sheep (*n* = 340)^1^.

**Variable**	**OR**	**95% CI**	**z-value**	***p*** **-Value**	**Relative importance**
(Intercept)	0.83	0.16–4.30	0.22	0.82	
Farm size: < 150 sheep	0.43	0.11–1.63	1.23	0.21	0.75
Farm size: 150–500 sheep	0.76	0.30–1.90	0.59	0.56	
Farm size: > 500 Sheep (ref)					
Age category: Young animals	0.21	0.12–0.40	4.90	<0.001^**^	1
Age category: Adults (ref)					
Abattoir: 2—Campania	3.99	1.53–10.40	2.82	0.004^*^	1
Abattoir: 1—Latium (ref)					
Domestic cat density: More than 5 cats/km^2^	1.27	0.66–2.45	0.72	0.47	0.59
Domestic cat density: 1–5 cats/km^2^	0.73	0.33–1.66	0.74	0.46	
Domestic cat density: < 1 cats/km^2^ (ref)					
Slope: High	0.97	0.20–5.25	0.04	0.97	0.54
Slope: Medium	2.47	0.31–19.64	0.85	0.39	
Slope: Low	1.83	0.37–8.97	0.74	0.46	
Slope: Flat (ref)					
Distance from water bodies: more than 350	0.99	0.64–1.54	0.03	0.98	0.24
Distance from water bodies: 100–350 m	1.15	0.62–2.11	0.44	0.66	
Distance from water bodies: < 100 m (ref)					

1 Animals with missing information for the tested variables were not included in the model.

* <0.01

** <0.001.

The serological status of the single animals was the dependent variable, while the variable “farm” was included as a random effect. The reported results are a summary of the averaged odds ratios with 95% CI obtained from the top model set.

As regards the bioclimatic variables, we observed that 5 out of 19 of them were statistically associated with the presence of *T. gondii* antibodies (Table 1).

## 4. Discussion

The present study provides updated information regarding the *T. gondii* seroprevalence of sheep slaughtered in Central-Southern Italy and explored the possibility that animal-related, environmental, and, for the first time in this country, bioclimatic factors can influence the likelihood of infection.

The results concerning the detection of *T. gondii* antibodies were consistent with those of several Italian surveys conducted during the last 15 years and confirmed that the seropositive rate in the sheep population is relevant. Although there were numerous differences in the study design and analytical methods, the *T. gondii* seropositivity of sheep continues to be average, around 50%, with most of the studies reporting percentages ranging between 40 and 65% (5–10). In contrast, *T. gondii* seroprevalence in other domestic species such as pigs has significantly decreased in most parts of the world over the years (19–21). This could be explained by the fact that sheep farming, contrary to those of other domestic species, continues to be mainly conducted outdoors; therefore, it is difficult to implement those biosecurity measures that permit the reduction in contact occasions with infectious forms of the parasite. This trend might be relevant in terms of public health; pork products have been traditionally considered the most relevant species regarding the risk of human toxoplasmosis through meat consumption but lamb or mutton might have an increasing importance over the next few years (22).

To our knowledge, only two further studies investigated the presence of *T. gondii* antibodies in lambs/young animals in Italy, and the reported positivity rate was very close to the one shown by our research (respectively, 41.7 and 41.0%) (6, 10). As expected, we also found that young animals presented a significantly lower likelihood of being seropositive than adult sheep, with the former having fewer occasions of being exposed to *T. gondii* oocysts during their life. However, this difference is likely even larger, considering that our serological tests, as the aforementioned studies, were unable to discriminate between antibodies that were derived from active immunity (i.e., produced after a natural infection) and those acquired through passive immunity from the mother. The persistence of antibodies obtained through the colostrum in lambs is still the subject of debate but the accredited hypotheses estimate that these antibodies are detectable for up to a maximum of 4 months (23, 24).

In addition to the aforementioned difference between the age groups, the analysis of risk factors showed a significant variation in seroprevalence based on the abattoir of sampling. This can substantially depend on the completely different provenience of the animals as abattoir 1 slaughtered sheep from Tuscany and Latium, while abattoir 2 slaughtered sheep were from the remaining regions (Campania and Basilicata). It is well known that the occurrence of toxoplasmosis can differ on a geographical basis. The probability of acquiring infection for a sheep is due to a combination of different factors such as certain husbandry conditions in the farm of origin and/or given environmental factors. Indeed, several farm-related factors, many of which were not included in this study, such as the management of drinking water and feed, might be important. Acquiring these additional data was not within our capability since it is a resource-demanding activity that implied on-site inspections and/or questionnaire administration to the farmers (8, 25–28).

Many studies identified farm size as a risk factor for sheep toxoplasmosis, although conflicting results have been reported. In some cases, small herds have been associated with a higher seroprevalence rate; as these herds are usually managed traditionally, the possible causes indicated by the authors were a higher frequency of grazing, which implies more occasions of exposure to *T. gondii* and lack of biosecurity-hygienic measures that do not allow us to adequately limit the contact with cat feces (8, 29). In contrast, other studies reported that the seroprevalence was positively correlated with the farm size (9, 15, 26, 30). Persistence of *T. gondii* infections in large herds may be due to (1) an increased chance of contact with cat's feces due to the lower amount of available space in the pens or (2) a more demanding management that can fail to interrupt the cycle of the parasite (i.e., the use of a contaminated concentrate or the delayed removal of infectious materials) (15, 26, 30). The farm size proved to be a relevant variable in our top model set; however, our research did not show any statistically significant association between the farm size and the chance of infections. To reduce the uncertainty associated with the impact of the farm size, “*ad hoc*” studies should investigate the different husbandry dynamics/characteristics of large/small herds that play a major role in *T. gondii* infections.

Many researchers reported that the presence of cats was associated with a higher seroprevalence of *T. gondii* in de Moura et al. (12), Guo et al. (1), and Andrade et al. (25). For this reason, we explored the possibility of a relationship between the density of domestic cats in the farm area and the seropositivity rate. We observed that higher density values (more than 5 cats/km^2^) corresponded to higher percentages of infection, and the association with the seropositivity rate was found to be statistically significant by the univariate analysis (*p* = 0.006; Supplementary Table 1). The domestic cat density was also included as a predictor in most of the top model candidates, although it did not have a statistically significant effect. It is important to highlight that we did not directly measure the cat population in the studied areas but we obtained estimates correlating the human density with the presumptive number of domestic cats on the basis of a previous study performed in a similar rural territory (31). Therefore, we cannot exclude inaccuracy from the estimates for some areas. Moreover, due to the lack of available data on the presence of stray cats, it was not possible to assess the impact of the overall feline population on the risk. However, our results are plausible and tend to confirm the importance of domestic cats in *T. gondii* transmission in sheep (1, 3).

Environmental and climatic conditions can impact the spread and survival of the oocysts increasing the occasions of exposure for susceptible animals to the parasite (32, 33). Owing to their hydrophilic and scarcely adhesive surface, the oocysts can mobilize following heavy rainfalls and disperse in freshwaters (2). Abundant rainfalls may also contribute to river or stream flows which might be responsible for the transport of contaminants and favor their spread in the environment (34). Miller et al. (35), for example, found a positive relationship between sea otter *T. gondii* infection and sampling areas where a maximal freshwater runoff was observed (35). Afonso et al. (36) observed a higher seroprevalence in cats experiencing wet winter seasons for many years (36). Our findings tend to be consistent with this hypothesis as we observed a higher probability of cases in areas with a higher precipitation level, based on the analysis of bioclimatic variables (Table 1, BIO 15 and 19). On the contrary, our analysis showed that proximity to a running water source was weakly associated with the likelihood of infection, although other studies suggested that the presence of streams or rivers may favor the spread of oocysts in a certain area as well as contribute to a moist environment that supports the oocyst survival and provide food availability for the *T. gondii* transport hosts such as insects (32). However, the role of water sources and rainfall as risk factors for sheep toxoplasmosis is still not clear, as few studies that were focused on this species are available to date. For example, apart from the present study, to our knowledge, no studies have been published on the relationship between the distance from water sources and the seropositivity rate of domestic sheep. There was only one study, conducted in Greece, which studied the association between rainfall and *T. gondii* infection in sheep applying a similar methodology; even if the climate conditions were presumably similar to Italy, the researchers reported contradictory results, with a *T. gondii* seropositivity rate negatively associated with the level of precipitation during the driest and warmest periods (26). It is difficult to explain such a difference, considering that the high number of variables can impact oocysts' infectiveness and/or exposure. We can hypothesize that dissimilar local conditions not contemplated in the two studies (i.e., a certain kind of vegetation or unknown soil characteristics) might have played an important role in creating suitable environments for *T. gondii* survival or spread (i.e., favoring the moisture entrapment).

Three bioclimatic variables concerning the temperature range (BIO 2, BIO 3, and BIO 7) became significant risk factors in our study; sheep raised in farms where lower diurnal or annual temperature range values were registered had a lower likelihood of being seropositive. Temperature can affect the survival rate of *T. gondii* oocysts in the soil and, indirectly, impact the probability of animal infection (16, 37). The presence of oocysts in areas with lower temperature ranges may imply that oocysts are less exposed to extremely harmful temperatures, and consequently, their probability of being infectious when ingested by sheep is higher. Other studies have speculated that temperate climate conditions might favor the survival time of the oocysts (38, 39). However, Kantzoura et al. (26) did not find any association between *T. gondii* seroprevalence in sheep and temperature range after analyzing the same bioclimatic variables; meanwhile, they found that a higher temperature was a significant factor (26). As no other analogous studies concerning sheep or other species are available, our hypothesis needs to be verified by collecting additional information.

Despite the novel insights the present study provides into ovine toxoplasmosis, the mentioned limitations do not allow us to clearly elucidate several aspects that should be explored through future investigations. To better understand the epidemiology of *T. gondii*, the prevalence of the parasite and risk factors should be studied with large-scale surveys that involve an accurate collection of information concerning farm characteristics and management aspects. The diagnostic techniques should be able to determine the real exposure to the parasite (i.e., detecting antibodies from passive immunity), and the evidence of infection should be considered in relation to a more comprehensive climate and an environmentally big dataset to identify those conditions that impact on the complex *T. gondii* cycle.

## 5. Conclusion

The occurrence of *T. gondii* infection remains relevant in the Italian sheep population, and considering the zoonotic potential of the parasite, this evidence needs to be carefully considered for its public health implications. Some specific environmental or climate conditions appear to favor the diffusion of sheep toxoplasmosis; this type of information could be used to design risk-based control plans in the future, but further investigations should be conducted to achieve a more consolidated knowledge in this field.

## Data availability statement

The raw data supporting the conclusions of this article will be made available by the authors, without undue reservation.

## Ethics statement

The animal study was reviewed and approved by Commissione ricerche dell'Istituto Zooprofilattico Sperimentale del Lazio e Toscana.

## Author contributions

RC and PR designed the survey. RC, ST, SS, and AB collected the samples which were tested by DS and ES. ES supervised the analytical activities and provided assistance for the manuscript. PR was responsible for data collection and RP created the GIS. RC performed data analysis and wrote the article with RP collaboration. All authors read and approved the final manuscript.
