# Supplementary material for: Toxoplasma gondii in sheep: Serological occurrence at slaughterhouse level in Italy and environmental risk factors
Source: Front Vet Sci. 2023 Mar 23;10:1057277. doi: 10.3389/fvets.2023.1057277 (PMC10076628; doi:10.3389/fvets.2023.1057277)
Supplement: Supplementary file 1 [file Table_1.DOCX]

**Supplementary materials**

Table S1 - *T. gondii* seropositive rate by animal-related and environmental factors (univariate analysis)

| **Characteristic** | **Negative,** N=182^1^ | **Positive,** N=218^1^ | **p-value^2^** | |
| --- | --- | --- | --- | --- |
| **Farm size** |  |  | 0.35 | |
| > 500 sheep | 63 (50%) | 62 (50%) |  | |
| < 150 sheep | 61 (47%) | 68 (53%) |  | |
| 150 - 500 sheep | 46 (41%) | 66 (59%) |  | |
| Not available | 12 | 22 |  | |
| **Abattoir** |  |  | 0.002 | |
| Abattoir 1- Latium | 100 (54%) | 86(46%) |  | |
| Abattoir 2 - Campania | 82(38%) | 132(62%) |  | |
| **Age category** |  |  | <0.001 | |
| Adults | 78 (37%) | 135 (63%) |  | |
| Lambs/Young animals | 104 (56%) | 83 (44%) |  | |
| **Land Cover Class** |  |  | 0.18 | |
| Agricultural surface | 161 (46%) | 191 (54%) |  | |
| Artificial surfaces | 12 (44%) | 15 (56%) |  | |
| Forest and Seminatural areas | 9 (56%) | 7 (44%) |  | |
| Water bodies | 0 (0%) | 5 (100%) |  | |
| **Altitude** |  |  | 0.10 | |
| Mountainous (above 700 amsl) | 26 (57%) | 20 (43%) |  | |
| Plain (0-200 amsl) | 40 (51%) | 38 (49%) |  | |
| Semi-mountainous (200-700 amsl) | 116 (42%) | 160 (58%) |  | |
| **Slope** |  |  | 0.28 | |
| Flat | 23 (45%) | 28 (55%) |  | |
| High | 9 (56%) | 7 (44%) |  | |
| Low | 127 (47%) | 141 (53%) |  | |
| Medium | 23 (35%) | 42 (65%) |  | |
| **Aspect** |  |  | 0.077 | |
| East | 28 (44%) | 36 (56%) |  | |
| North | 13 (46%) | 15 (54%) |  | |
| North-East | 12 (36%) | 21 (64%) |  | |
| North-West | 13 (32%) | 28 (68%) |  | |
| South | 53 (59%) | 37 (41%) |  | |
| South-East | 22 (47%) | 25 (53%) |  | |
| South-West | 15 (43%) | 20 (57%) |  | |
| West | 21 (58%) | 15 (42%) |  | |
| Not available | 5 | 21 |  | |
| **Distance from water sources (meters)** |  |  | 0.14 | |
| Less than 100 m. | 59 (49%) | 61 (51%) |  | |
| 100-350 m. | 64 (40%) | 98 (60%) |  | |
| More than 350 m. | 59 (50%) | 59 (50%) |  | |
| **Soil composition** |  |  | 0.035 | |
| Clay | 20 (69%) | 9 (31%) |  | |
| Others | 99 (42%) | 138 (58%) |  | |
| Sand | 22 (52%) | 20 (48%) |  | |
| Sand clay | 41 (45%) | 51 (55%) |  |  |
| **Domestic cat density** |  |  | 0.006 |  |
| < 1 cats/km^2^ | 83 (47%) | 92 (53%) |  |  |
| 1-5 cats/km^2^ | 45 (58%) | 32 (42%) |  |  |
| More than 5 cats/km^2^ | 54 (36%) | 94 (64%) |  |  |
| ^1^ n (%) |  |  |  | |
| ^2^ Pearson's Chi-squared test, Fisher's exact test | | |  | |
